# The Mutually Mediated Chloride Intracellular Channel Protein 1 (CLIC1) Relationship between Malignant Cells and Tumor Blood Vessel Endothelium Exhibits a Significant Impact on Tumor Angiogenesis, Progression, and Metastasis in Clear Cell Renal Cell Carcinoma (ccRCC)

**DOI:** 10.3390/cancers14235981

**Published:** 2022-12-03

**Authors:** Adela Maria Ferician, Ovidiu Catalin Ferician, Alexandru Nesiu, Andrei Alexandru Cosma, Borislav Dusan Caplar, Eugen Melnic, Anca Maria Cimpean

**Affiliations:** 1 Doctoral School in Medicine, Victor Babes University of Medicine and Pharmacy, 300041 Timisoara, Romania; 2 Department of Orthopedy and Traumatology/Urology, Victor Babes University of Medicine and Pharmacy, 300041 Timisoara, Romania; 3 Department of Urology, Faculty of Medicine, “Vasile Goldiș” Western University, 310025 Arad, Romania; 4 Department of Microscopic Morphology/Histology, Victor Babes University of Medicine and Pharmacy, 300041 Timisoara, Romania; 5 Department of Pathology, “Nicolae Testemitanu” State University of Medicine and Pharmacy, 2004 Chișinău, Moldova; 6 Angiogenesis Research Center, Victor Babes University of Medicine and Pharmacy, 300041 Timisoara, Romania; 7 Center of Expertise for Rare Vascular Disease in Children, Emergency Hospital for Children Louis Turcanu, 300041 Timisoara, Romania

**Keywords:** clear cell renal cell carcinoma (ccRCC), chloride intracellular channel protein 1 (CLIC1), tumor angiogenesis, TNM, metastasis

## Abstract

**Simple Summary:**

Tumor cells interact with endothelial cells via chloride intracellular channel protein 1 (CLIC1) micro vesicles transfer, inducing endothelial cell proliferation, migration, and tube formation, according to in vitro studies. Furthermore, CLIC1-positive tumor cells appear to have a high invasive and metastasis potential in vitro, but data on human tumor tissues are extremely scarce. CLIC1 expression in tumor blood vessels is reported for the first time here, suggesting CLIC1 as a new endothelial marker. CLIC1 co-expression in tumor and endothelial cells stratified ccRCC cases into several subgroups, and this co-expression influences TNM staging parameters. Based on these findings, we can conclude that CLIC1 is important in angiogenesis, tumor progression, and metastasis in ccRCC. CLIC1 inhibition may have a synergistic effect on tumor cells, as well as tumor vasculature, as our team previously reported for a ccRCC patient-derived tumor xenograft implanted on a chick embryo chorioallantoic membrane treated with anti-CLIC1 antibodies.

**Abstract:**

Background: Overexpression of chloride intracellular channel protein 1 (CLIC1) in tumor cells has been confirmed, but it has received less attention in the tumor blood vessel endothelium. Aim: The assessment of CLIC1 expression in ccRCC tumor blood vessels and its relationship with TNM parameters and tumor cell CLIC1 expression. Methods: CLIC1 immunostaining in ccRCC was evaluated in 50 cases in both malignant cells and tumor blood vessels (CLIC1 microvessel density-CLIC1-MVD) and was correlated with TNM staging parameters. Results: CLIC1-MVD was observed in approximately 65% of cases, and CLIC1 co-localization in both tumor and endothelial cells was observed in 59% of cases. ccRCC was classified into four groups (Classes 0–3) based on the percentage of positive tumor cells, with each group including sub-groups defined by CLIC1 expression in the endothelium. Class 3 (60–100% positive tumor cells) had the highest CLIC1-MVD, with an impact on T and M parameters (*p* value = 0.007 for T, and *p* value = 0.006 for M). For cases with CLIC1 intracellular translocation, there was a strong correlation between CLIC1-MVD and M (*p* value < 0.001). Conclusions: Co-expression of ccRCC tumor and endothelial cells promotes tumor progression and metastasis and should be investigated further as a potential therapeutic target for ccRCC and other human malignancies.

## 1. Introduction

Although tumor angiogenesis is not a new concept [1] it is still poorly understood. The heterogeneity of endothelial cells lining the tumor blood vessels (BVs) remains the most difficult issue to explain in the development of tumor neo-vessels. There is not yet a described specific endothelial marker exclusively expressed in tumor endothelium that could be used as a therapeutic target for drugs targeting tumor neo-vessels, but not normal vasculature. To increase the efficiency of future targeted therapies, the side effects of current antiangiogenic and anti-vascular medications require the identification of new specific markers for tumor vessel endothelium and perivascular cells [2]. Several tumor endothelial-specific indicators for malignancies such as colon cancer [3] and breast cancer [4] have been proposed. However, based on these present findings, no effective anti-vascular and/or antiangiogenic drugs have yet been produced [5].

Only a few markers are expressed in tumor BVs endothelial cells, but not in quiescent normal vascular endothelial cells. Endoglin (CD105) is a protein produced in activated endothelial cells during inflammatory and tumoral settings, and it has therefore been utilized to assess tumor angiogenesis in a variety of premalignant [6] and malignant [7,8,9,10,11] conditions. Several preclinical and clinical trials [12,13,14,15] reported the utility of anti-endoglin antibody-based therapy TRC105 alone or in combination with other antiangiogenic or anti-vascular therapies.

TRC105 anti-endoglin treatment in conjunction with bevacizumab [16] has been shown to improve renal cancer responsiveness; however, the outcomes have been mixed. Another study recently published the findings of a Phase I clinical trial involving TRC105 and axitinib as treatment for individuals who had failed anti-VEGF medication alone. TRC105-axitinib appears to target cancer stem cells that express CD105, which are known to facilitate the resistance to VEGF pathway inhibitors [17]. The findings of the previous study are encouraging because of the long-term activity in a VEGF inhibitor-resistant population and the alteration of various angiogenic indicators [17].

Except for endoglin, which is used to highlight activated tumor BVs in renal cell carcinomas (in conjunction or not with other common endothelial markers) [18,19,20], there is sporadic data on B7-H3 [21] or endocan [22] expression in newly formed blood vessels from renal cell carcinomas, but there is no validated data on their use as therapeutic targets.

To develop new targeted therapeutics for renal cell carcinomas, it is necessary to uncover and evaluate new endothelium markers.

In malignant disease, CLIC1 appears to be a prospective therapeutic target for both tumor and endothelial cells.

Our research previously evaluated chloride intracellular channel 1 (CLIC1) in ccRCC, indicating that it may stratify ccRCC based on its expression pattern, as well as its differential expression in relation to tumor grade [23]. We discovered that CLIC1 was expressed not only by ccRCC tumor cells, but also by endothelial cells lining intratumor and peritumor blood vessels with a shape very suggestive of neo-vessels.

Only a few articles [24,25] indicate CLIC1 expression in tumor BVs endothelium, and none mention CLIC1 expression in neo-vessels from clear cell renal cell carcinomas (ccRCC).

We thought it would be interesting to examine CLIC1 expression in tumor BVs endothelium from ccRCC and discuss its impact on tumor progression, based on recent findings in the literature regarding the vesicular transfer of micro vesicles from glioblastoma malignant cells to microvascular endothelial cells [26].

## 2. Materials and Methods

The current study began after all ethics committee approvals from Vasile Goldis University/Faculty of Medicine Arad, Romania (No. 91/19.07.2018) had been obtained.

### 2.1. Patients and Biopsies

The study used formalin-fixed paraffin embedded (FFPE) specimens from 60 cases of clear cell renal cell carcinoma diagnosed between 2005 and 2015. None of the patients in the study received treatment prior to surgery. Two independent pathologists reviewed the corresponding hematoxylin and eosin stained slides to confirm the initial histopathology diagnosis and to evaluate tissue quality, which is required for proper case selection for immunohistochemistry. The tissue quality was evaluated by performing an immunostaining for vimentin (clone V9). Cases positive for vimentin (in the tumor stroma) were considered suitable for selection and for the future immunohistochemical procedures. Out of the initial 60 cases, 50 met the quality standards for immunohistochemistry.

### 2.2. Immunohistochemistry of CLIC1 

Immunohistochemistry of CLIC1 was performed on three-micrometer thick sections, in each case. The slides were loaded into the Bond Max Autostainer (Leica Microsystems, Leica Biosystems Newcastle Ltd., Newcastle upon Tyne, UK), and a predefined program was chosen to complete the CLIC1 immunostaining protocol. Monoclonal mouse-anti-human CLIC1 antibodies IgG2a (100 g/mL, clone 356.1, code sc-81873, Santa Cruz Biotechnology, dilution 1:200) were incubated for 30 min at room temperature, using a biotin-free Bond Polymer Refine Detection System (Leica Biosystems, Newcastle Ltd., Newcastle upon Tyne, UK), which included a 3, 3 diaminobenzidine-based visualization step and nuclear counterstaining with hematoxylin. CV Mount (mounting medium from Leica Biosystems, Newcastle Ltd., UK) was used as a permanent mounting medium for immunohistochemistry-stained slides.

### 2.3. Acquisition of Images and Data Interpretation

Stained slides were automatically scanned with a Panoramic Desk Digital Scanner (3D Histech, Budapest, Hungary), archived in the Case Center, and viewed with Case Viewer, a software packages from 3D Histech, Budapest, Hungary, which comes with the optical system and allows for an overview of the microscopic elements in the area of interest. The WHO Classification of Renal Tumors criteria were used to evaluate hematoxylin and eosin stained slides. CLIC1 immunoexpression was measured in tumor blood vessels, as well as tumor cells. CLIC1 expression in tumor cells was quantified in relation to expression pattern (nuclear-N/cytoplamic-C/membranar-M or combined-NC/NMC) due to its well-known high ability for translocation between the nucleus/cytoplasm and the membrane. CLIC1-positive cases were classified not only by the immunoexpression pattern, but also by the percentage of CLIC1-positive tumor area, as follows: class 0-0—10% of CLIC1-positive tumor cells, class 1-10—30% of tumor cells positive for CLIC1, class 2-30—60% of tumor cells positive for CLIC1, and class 3-60—100% CLIC1-positive tumor cells. The number of CLIC1-positive tumor blood vessels was determined using hot spot methods for assessing microvessel density (MVD). The TNM staging parameters were correlated with the microscopic data.

### 2.4. Statistical Analysis

JAMOVI software for macOS devices was used for statistical analysis. A statistical correlation was assessed and considered significant for a *p* value of 0.05 or less.

### 2.5. CLIC1 Role and Expression in ccRCC and Bioinformatic Analysis 

CLIC1 role and expression in ccRCC and bioinformatic analysis was performed by the assessment of Human Protein Atlas databases to highlight CLIC1 expression in normal renal tissue and corresponding malignant tissues from ccRCC. Then, The Cancer Genome Atlas (TCGA) databases was accessed by using the cBioPortal website (www.cbioportal.org, accessed on 16 November 2022) to find details related to the role and expression of CLIC1in ccRCC.

## 3. Results

### 3.1. ccRCC Cases Classification according to CLIC1 Expression in ccRCC Tumor Cells and Tumor Blood Vessels Endothelium

CLIC1 expression was measured not only in tumor cells (ccRCC-TC), but also in the tumor blood vessel endothelium (CLIC1 microvessel density, ccRCC-TBvsE). CLIC1-positive tumor cells with homogeneous or heterogeneous distribution within the tumor area were found in 87.5% of ccRCC cases. CLIC1 was found at the endothelial level in approximately 65% of cases, and 59% of cases had CLIC1 co-localization in both tumor cells and tumor blood vessel endothelium (Table 1).

CLIC1 expression in tumor cells stratified in ccRCC cases according to 4 classes. The percentage of cases for each class related to its expression in the tumor blood vessels is detailed in Table 2.

CLIC1 expression in tumor blood vessel endothelium was present in more than half of the cases (59%), and the majority of them (39%) were classified as class 3 due to high levels of CLIC1 expression in the tumor cells.

### 3.2. Statistical Analysis of Correlation between Different Subgroups to the pTNM Staging Parameters

Based on previous findings of CLIC1 co-expression heterogeneity in tumor and endothelial cells, we decided to investigate whether there are any correlations between this expression and the *p* TNM parameters. The number of ccRCC cases distributed according to the interrelation between pTNM/ccRCC classes and CLIC1 expression in tumor blood vessel endothelium and tumor cells (Table 3).

#### 3.2.1. Assessment of Class 0 Cases Did Not Show Any Significant Correlation in between TNM Parameters and CLIC1-MVD

**Figure 1:**
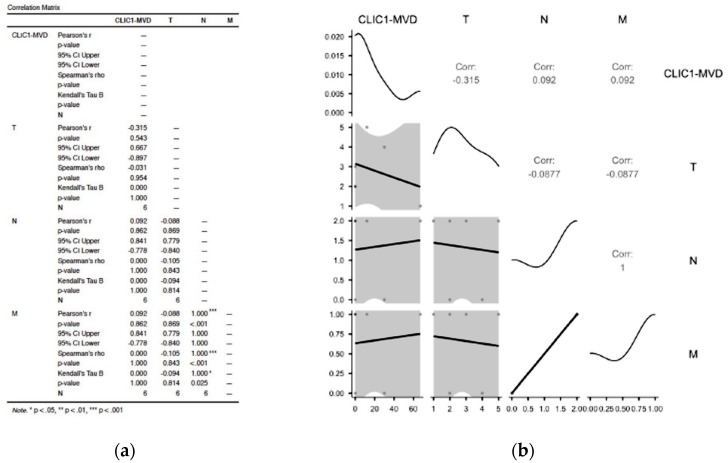
Statistical analysis results (**a**) and correlation plot (**b**) for Class 0 cases related to the TNM staging parameters. No significant correlation was recorded between CLIC1-MVD and T, N, or M parameters. A strong significant correlation has been observed between the N and M parameters, suggesting that CLIC1-MVD may favor nodal and distant metastasis.

Class 0 cases accounted for 12% of all cases. CLIC1-positive tumor blood vessels were found in half of them, with densities ranging from 13 to 67 vessels per microscopic field X20. There was no significant correlation between CLIC1-MVD and TNM staging parameters in this class (*p* value = 0.543 for T, *p* value = 0.862 for N, and *p* value = 0.862; Figure 1a,b).

#### 3.2.2. Analysis of Cases Grouped as Class1 Have Shown no Significant Influence on TNM Staging Parameters

**Figure 2:**
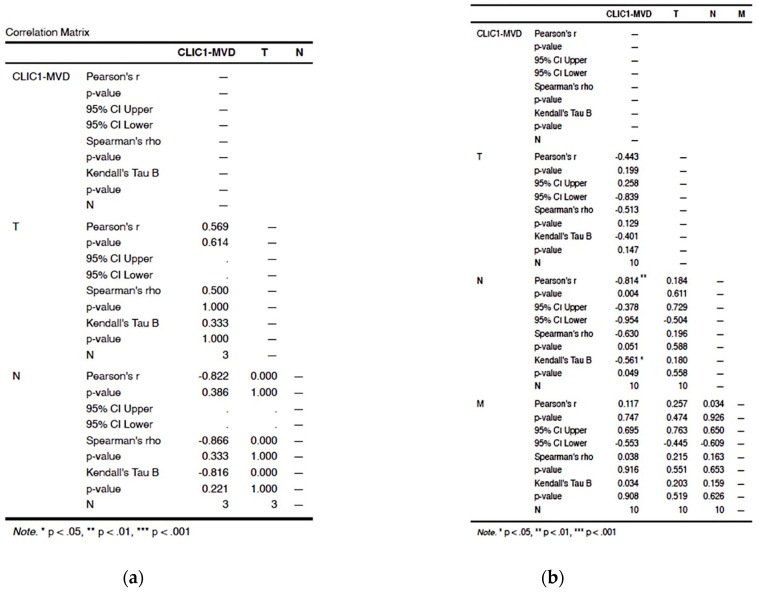
Correlation matrix from statistical analysis applied for Class 1 (**a**) and Class 2 (**b**) ccRCC subgroups related to CLIC1 expression in tumor blood vessels (CLIC1-MVD). Note the lack of significant correlation for Class 1 (**a**) and the significant correlation between CLIC1-MVD and nodal status N from TNM staging for Class 2 of ccRCC.

Class 1 exhibited the fewest cases, but they all expressed CLIC1-positive tumor blood vessels, with densities ranging from 10 to 17 vessels/microscopic field X20. According to statistical analysis, the presence of CLIC1 did not influence TNM staging in Class 1 (*p* value = 0.614 for T, and *p* value = 0.386 for N; Figure 2a).

#### 3.2.3. Increased CLIC1 Expression in ccRCC Tumor Cells May Influence Nodal Metastasis

For Class 2, CLIC1 expression in tumor blood vessels was significantly inversely correlated with the N parameter from the TNM staging system (*p* value = 0.004, Figure 2b).

#### 3.2.4. CLIC1 Expression Was Highest for Class 3 Subgroup Tumor Cells and Significantly Influenced CLIC1-MVD, Tumor Stage, Nodal and Distant Metastasis

**Figure 3:**
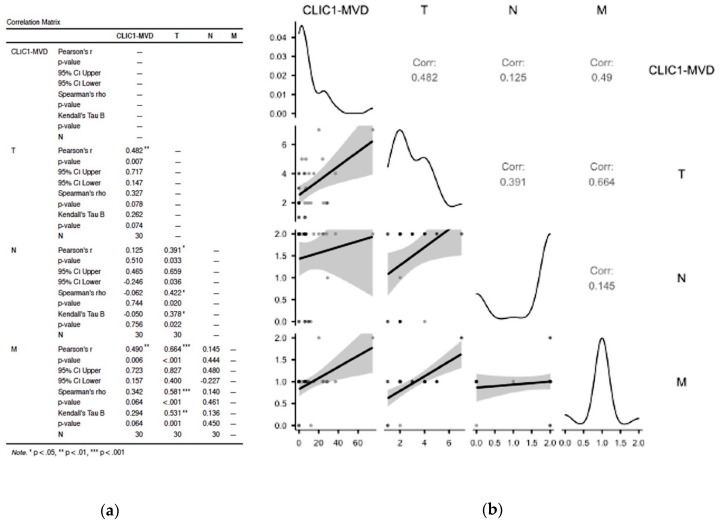
Correlation between TNM staging parameters and CLIC1-MVD for the Class 3 group ccRCC cases. Correlation matrix (**a**) highlighted significant correlation between CLIC1-MVD, T, and M parameters. Moreover, Class 3 is the only ccRCC subgroup where the T parameter is strongly correlated with the N and M parameter, suggesting that a high CLIC1 expression in tumor cells may favor nodal and distant metastasis. Graphical representation of the data from the correlation matrix is found in (**b**).

The Class 3 subgroup of cases had the greatest number of CLIC1-positive tumor blood vessels. Increased CLIC1-MVD in Class 3 cases had an impact on T and M parameters (*p* = 0.007 for T and *p* = 0.006 for M). Significant correlations were also found between TNM staging parameters. T and N parameters were significantly correlated (*p* value = 0.033), but the strongest correlation was observed between T and M parameters (*p* value = 0.001, Figure 3a,b)).

### 3.3. The ccRCC Assessment According to CLIC1 Expression Pattern in Tumor Cells, Its Expression in Tumor Blood Vessels, and TNM Staging Parameters

CLIC1 has a high ability to translocate between three major cellular compartments (nucleus, cytoplasm, and membrane). CLIC1 nuclear translocation is responsible for the increase in the malignant phenotype in tumor cells, in some cases. In our study, we discovered a high degree of variability in CLIC1 translocation in ccRCC tumor cells. This variability was examined in relation to the CLIC1-MVD and TNM parameters.

**Figure 4:**
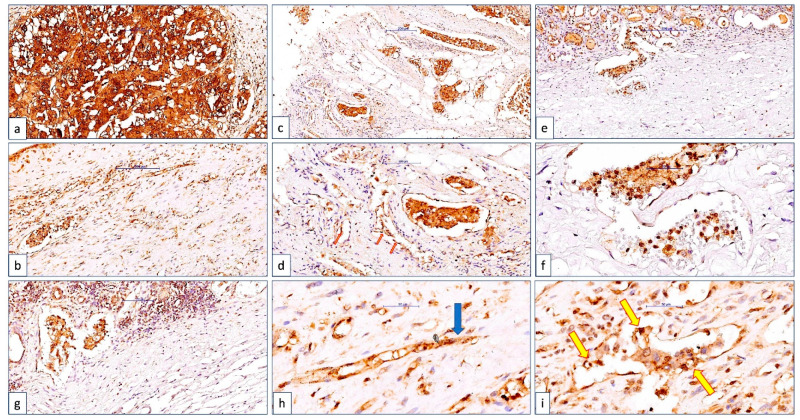
CLIC1 expression overview in a ccRCC case classified as pT4NxM1L1V1. The tumor area was strongly positive for CLIC1, with an NMC pattern (**a**). We detected intravascular CLIC1-positive tumor emboli in both small (**b**) and large vessels around the tumor (**c**,**d**), but also in capsular and subcapsular vessels of the adjacent kidney parenchyma far from the tumor (**e**,**f**). Large and small vessels of the endothelium lining were also positive for CLIC1. A particular aspect of CLIC1-positive tumor blood vessels was observed related to their morphology. CLIC1-positive intravascular pillars (**d**, red arrows), along with CLIC1-positive endothelial cell cords, which are highly suggestive of angiogenic sprouts (**h**, blue arrow), indicated a high angiogenic process around the tumor. A frequent phenomenon observed for CLIC1-positive cases, especially those with pT3a, 3b, and 4 regarding the angiogenic tumor vessels, was the interrelationship between intravascular emboli tumor cells, which were often interconnected with endothelial cells. Tumor cells and endothelial cells delineated spaces mimicking a new vascular luminal structure inside the existing vessels (**i**, yellow arrows). Capsular and subcapsular vessels containing CLIC1 tumor emboli were often associated with inflammation in the proximity (**g**). The most CLIC1-positive tumor blood vessels were found in the pT4 cases (75 vessels/X20 microscopic field; Figure 5). Scale bar: 200 μm for images (**a**,**c**,**e**); 100 μm for images (**b**,**d**,**f**); 50 μm for images (**g**,**h**,**i**).

CLIC1 was found in all three cellular compartments of tumor cells in 24% of cases (nuclear-N, cytoplasm-C, and membrane-M). This is known as the NMC subgroup, previously described by our team [23]. More than half of the cases in this group were classified as pT2b, 3a, 3b, or 4. An extensive lympho-vascular invasion was observed in the case staged as pT4 from the NMC group. The lymphatic and vascular emboli were found to be NMC-CLIC1-positive, as were the tumor cells. The endothelium of the peritumor blood vessels expressed CLIC1, with nuclear and cytoplasmic colocalization, as shown in Figure 4. This finding suggests that CLIC1 translocation is induced, not only in tumor cells, but also in the endothelium of tumor blood vessels. CLIC1-NMC-positive tumor emboli inside blood vessels with a CLIC1-positive endothelium have been observed far from the tumor in the subcapsular area.

**Figure 5:**
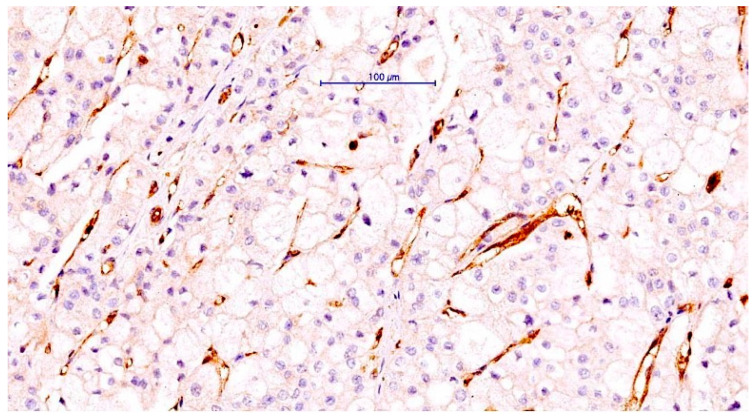
CLIC1 positivity inside the intratumor blood vessels endothelium in a ccRCC case with CLIC1-negative tumor cells. Note the high CLIC1-positive microvessel density and also the heterogeneity of their morphology. Scale bar: 100 μm.

**Figure 6:**
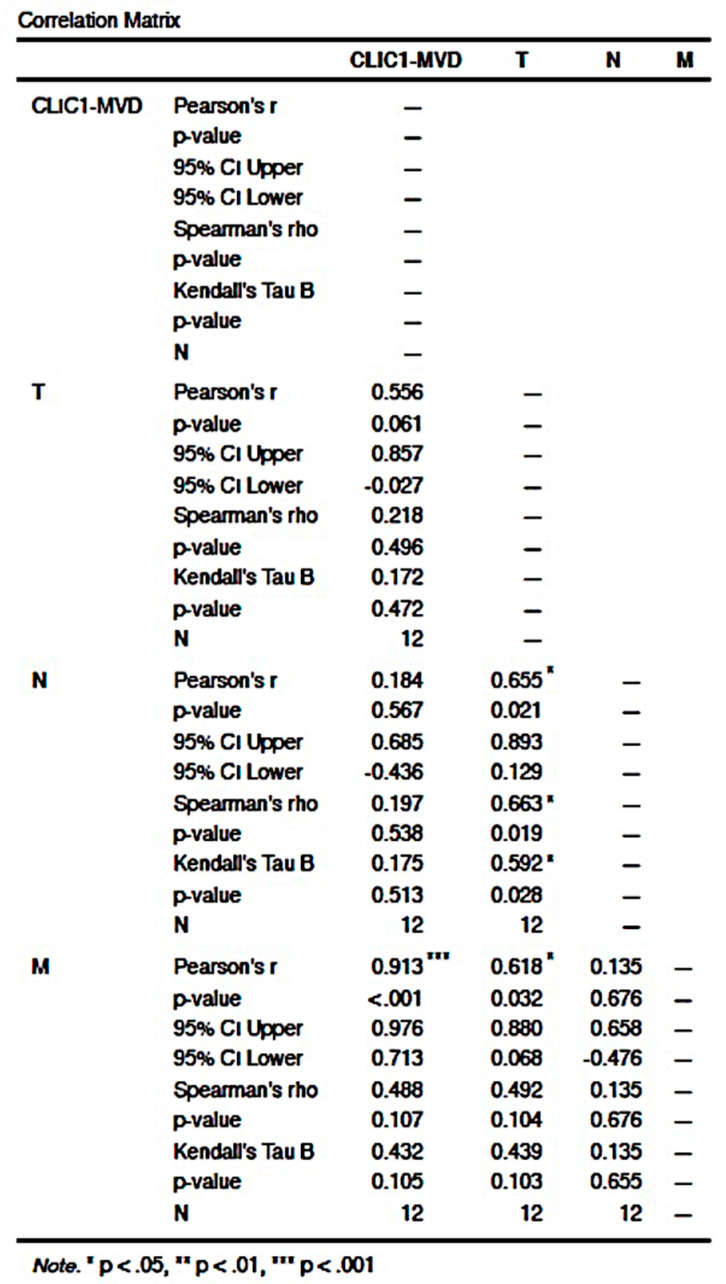
Correlation plot for the NMC subgroup related to CLIC1-MVD and the TNM staging parameters.

The CLIC1-NMC subgroup statistical analysis revealed a significant correlation between the CLIC1-MVD and the M parameter (*p* value < 0.001). The T parameter also had a significant correlation with N (*p* value = 0.021) and M (*p* value = 0.032, Figure 6). We found no significant correlation between CLIC1-MVD and the TNM staging parameters in any of the other pattern expression subgroups (NC, NM, C, N, M).

### 3.4. Analysis of Human Protein Atlas and the Cancer Genome Atlas Data Related to CLIC1 Expression in ccRCC and the Tumor Endothelium

Analysis of Human Protein Atlas and The Cancer Genome Atlas data related to CLIC1 expression in ccRCC and the tumor endothelium revealed that CLIC1, along with CLIC6, are reported to be expressed in both normal and ccRCC human kidney tissues. We analyzed CLIC1 expression (low or high) in ccRCC and found that it has an impact on patient survival, depending on the TNM staging parameters. We identified a total number of 528 cases with ccRCC from the TCGA databases. Survival analysis was based on low versus high CLIC1 mRNA expression in correlation with the tumor stage.

The global analysis of all 528 patient samples revealed that 35.7% of the total patients from the TCGA databases exhibited a high CLIC1 mRNA expression. Significant differences regarding survival were registered between cases with low and high CLIC1 expression, dependent by tumor stage and sex.

**Figure 7:**
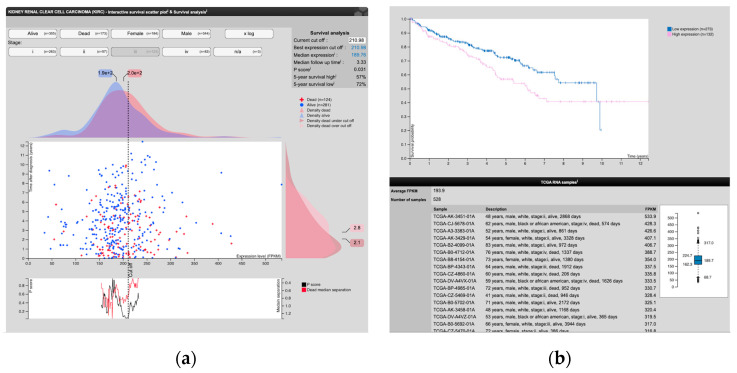
Comparative assessment of 5-year survival rate between ccRCC from TCGA database patients with high and low CLIC1 expression. The survival scatter plot (**a**) and the Kaplan–Meier survival curve (**b**) for stage III ccRCC showed significant survival differences related to CLIC1 expression levels. Note that high CLIC1 expression significantly reduces the 5-year survival rate (**b**) according to the Kaplan–Meier curve.

Cases with tumor stage III showed significant differences in the 5-year follow-up survival rate between CLIC1 high and low expressing cases. The 5-year survival rate was 57% for tumor stage III cases with high CLIC1 expression compared to a 72% 5-year survival rate in the case of low expressing patients from similar tumor stages (III) (*p* = 0.031; Figure 7b).

The analysis of the CLIC1 gene mutations in cases from TCGA revealed only one ccRCC case (0.39%) with a CLIC1 gene mutation; thus, further gene analysis could not be performed.

## 4. Discussion

Members of the intracellular chloride channel protein family have been extensively studied in recent years in relation to their overexpression in tumor cells with aggressive metastatic behavior [26,27]. CLIC1 overexpression in tumor cells has recently been identified as a potential angiogenic factor favoring tumor angiogenesis [28], invasion, and metastasis [29,30,31]. Cosnita et al. used anti-CLIC1 antibodies to demonstrate CLIC1-expressing tumor cell necrosis and tumor blood vessel inhibition on chick embryo chorioallantoic membrane ccRCC tumor model xenografts [32]. All 13 papers reporting CLIC1 expression in endothelial cells use only in vitro data [33].

The current study was conducted solely on human tumor specimens obtained from patients with ccRCC malignancy, and it is the first to report CLIC1 expression in human endothelial cells. We confirmed previous in vitro findings regarding CLIC1 expression at endothelial levels in these specimens.

Few studies have suggested that CLIC1-positive tumor cells communicate with endothelial cells by releasing CLIC1 extracellular vesicles with affinity for different endothelial cell receptors, such as TRMP7, for glioblastoma cells [25] or colocalize with CLT1 on angiogenic tumor blood vessels [34].

Evidence of a link between CLIC1-positive tumor cells and tumor blood vessel endothelial cells has grown in recent years, but CLIC1 expression in endothelial cells has only been reported on cultured endothelial cells [35]. CLIC1 was found to be expressed, not only in ccRCC tumor cells, but also in tumor blood vessel endothelial cells, suggesting that it could be used as a new potential endothelial marker for tumor angiogenesis assessment.

CLIC1′s angiogenic role has been reported in four papers, primarily using in vitro models [24,35] and, to a lesser extent, mice experimental models [36]. In our study, Class 3 ccRCC tumors with the highest CLIC1 expression in tumor cells contained the most CLIC1-positive tumor blood vessels. The current study found a correlation between CLIC1 expression in ccRCC malignant cells and CLIC1-MVD value, which supports the angiogenic role of CLIC1 suggested by previous in vitro studies. The role of CLIC1 in tumor progression and metastasis is well established [37].

In terms of CLIC1 expression in the blood vessel endothelium, our team reported its variability in the inflammatory triggered angiogenic endothelium from non-malignant conditions, such as rheumatoid and psoriatic arthritis, where vascular CLIC1 expression was highly influenced by therapy, as well as by the interrelationship with other stromal components [38]. For the present study, the capsular and subcapsular vessels with tumor emboli inside were frequently associated with an extensive inflammatory infiltrate, sometimes organized as a tertiary lymphoid tissue. Based on the preliminary findings from this study, CLIC1′s role as an angiogenic factor in human malignancies should be further investigated in other tumor types and correlated with other prognostic and survival factors.

Significant correlations between CLIC1-MVD and TNM staging parameters, as well as the microscopic presence of CLIC1-positive tumor emboli inside tumor blood vessels lined by a CLIC1-positive endothelium, support the angiogenic role of CLIC1 secreted by the tumor cells, while also confirming its involvement as a promoter of cancer progression and metastasis.

From the survival rate analysis performed for ccRCC cases found in TCGA database related to their CLIC1 expression, we found a total of 35.7% with high CLIC1 expression. Independently, we reported a group called Class 3, which includes 39% of cases with high CLIC1 expression in both tumor and endothelial cells. As is shown, the percentages from both analyses are mostly overlapping, giving strong evidence of CLIC1 involvement in tumor progression and metastasis.

In the present study, the highest CLIC1 expression in tumor cells and intravascular tumor emboli, along with an active angiogenic process demonstrated by tumor blood vessel morphology heterogeneity, was reported for tumor stage III. Survival analysis of TCGA databases showed that almost half of the ccRCC patients with high CLIC1 mRNA expression had died before the 5-year follow-up.

## 5. Conclusions

CLIC1 is heterogeneously expressed in ccRCC tumor cells and tumor blood vessel endothelium, influencing tumor stage and progression, as well as nodal and distance metastatic potential. Based on our previous experimental studies and on the findings of the present research, CLIC1 released by tumor cells and tumor emboli inside tumor blood vessels represent strong evidence of its angiogenic effect, supported by the presence inside the tumor and in peritumor area of small and well-defined blood vessels lining the CLIC1-positive endothelium, with a vascular morphology highly suggestive of an intense angiogenic process. CLIC1 expression in both tumor and endothelial cells mark CLIC1 as a potential dual target for specific antibodies-based therapy, which was proven to be effective in experimental models of ccRCC.

## Figures and Tables

**Table 1:** Percentage of CLIC1-positive cases according to its expression in tumor cells (ccRCC-TC), tumor blood vessels endothelium (ccRCC-TBvsE), or its co-localization in both tumor cells and tumor blood vessel endothelium (ccRCC-TC/ ccRCC-TBvsE).

CLIC1	% POSITIVE	% NEGATIVE
ccRCC TC	87.5	12.5
ccRCC TBvsE	65.11	34.88
ccRCC TC/ccRCCTBvsE	59	41

**Table 2:** ccRCC cases classified in 4 groups according to the percent of positive tumor cells and their subgroups stratification based on CLIC1 expression in the tumor blood vessel endothelium.

ccRCC Classes	Cases %	CLIC1-ccRCC TC-/ccRCC TBvsE-	CLIC1-ccRCC TC-/ccRCC TBvsE+	CLIC1-ccRCC TC+/ccRCC TBvsE-	CLIC1-ccRCC TC+/ccRCC TBvsE+
Class 0 (0–9%)	12%	6%	6%	0%	0%
Class 1 (10–29%)	6%	0%	0%	0%	6%
Class 2 (30–59%)	20%	0%	6%	0%	14%
Class 3 (60–100%)	62%	0%	0%	23.00%	39.00%
TOTAL	100%	6%	12%	23.00%	59.00%

**Table 3:** Number of ccRCC cases from each class reported in correlation with the CLIC1 TC/BvsE expression pattern corresponding to each of the pT stages, as well as the N and M parameters. Yellow highlighted columns point out negative cases. In this way, we may observe that the number of cases with CLIC1 co-expression in both tumor and endothelial cells increases from class1 to class 3, with most of the cases being evaluated as exhibiting pT 2a, 2b, 3a, 3b, and 4.

Classes	pTNM		CLIC1 TC/BvsE Expression Patterns			
			CLIC1-ccRCC TC-/ccRCC TBvsE-	CLIC1-ccRCC TC-/ccRCC TBvsE+	CLIC1-ccRCC TC+/ccRCC TBvsE-	CLIC1-ccRCC TC+/ccRCC TBvsE+
CLASS 0 12%	pT	1a	0	1	0	0
		1b	1	0	0	0
		2a	2	0	0	0
		2b	0	1	0	0
		3a	0	1	0	0
		3b	0	0	0	0
		4	0	0	0	0
	N	X	3	2	0	0
		0	0	1	0	0
		1	0	0	0	0
	M	X	3	2	0	0
		0	0	1	0	0
		1	0	0	0	0
CLASS 1 6%	pT	1a	0	0	0	0
		1b	0	0	0	1
		2a	0	0	0	1
		2b	0	0	0	0
		3a	0	0	0	0
		3b	0	0	0	0
		4	0	0	0	1
	N	X	0	0	0	1
		0	0	0	0	2
		1	0	0	0	0
	M	X	0	0	0	3
		0	0	0	0	0
		1	0	0	0	0
CLASS 2 20%	pT	1a	0	0	0	1
		1b	0	0	1	4
		2a	0	0	1	1
		2b	0	0	0	1
		3a	0	0	1	0
		3b	0	0	0	0
		4	0	0	0	0
	N	X	0	0	3	5
		0	0	0	0	1
		1	0	0	0	1
	M	X	0	0	2	6
		0	0	0	1	1
		1	0	0	0	0
CLASS 3 62%	pT	1a	0	0	2	2
		1b	0	0	5	6
		2a	0	0	2	1
		2b	0	0	0	3
		3a	0	0	0	5
		3b	0	0	0	0
		4	0	0	0	2
	N	X	0	0	9	13
		0	0	0	2	5
		1	0	0	0	1
	M	X	0	0	9	16
		0	0	0	2	0
		1	0	0	0	2

## Data Availability

Data is contained within the article.
